# A Long-Term Pilot Study on Sex and Spinal Cord Injury Shows Sexual Dimorphism in Functional Recovery and Cardio-Metabolic Responses

**DOI:** 10.1038/s41598-020-59628-6

**Published:** 2020-02-17

**Authors:** Adel B. Ghnenis, Daniel T. Burns, Wupu Osimanjiang, Guanglong He, Jared S. Bushman

**Affiliations:** University of Wyoming School of Pharmacy, 1000 East University Avenue, Department 3375, Laramie, WY 82071 USA

**Keywords:** Neuroscience, Cardiovascular biology, Metabolism

## Abstract

More than a quarter of a million individuals in the US live with spinal cord injury (SCI). SCI disrupts neural circuitry to vital organs in the body. Despite severe incidences of long-term peripheral complications from SCI, the cardio-metabolic consequences and divergences in sex-related responses are not well described. We examined the effects of SCI on functional recovery, cardiac structure and function, body composition, and glucose metabolism on adult female and male Sprague Dawley (SD) rats. SCI was induced at T10 via contusion. Measured outcomes include behavioral assessment, body weight, dual-energy X-ray absorptiometry (DEXA) for body composition, echocardiography for cardiac structure and function, intraperitoneal glucose tolerance test (IPGTT) for glucose metabolism, insulin tolerance test (ITT), and histology of cardiac structure at the endpoint. There was a decrease in body fat percentage in both sexes, with SCI females disproportionately affected in percent body fat change. Left ventricular internal diameter during systole (LVIDs) was decreased in SCI females more than in SCI males. No significant differences in glucose metabolism were observed up to 20 weeks post-injury (PI). These data show significant cardio-metabolic differences as a consequence of SCI and, furthermore, that sex is an underlying factor in these differences.

## Introduction

Traumatic spinal cord injury (SCI) represents a major public challenge worldwide^1^. Individuals with SCI are known to face lifelong disability that results from ineffective motor regeneration and aberrant sensory reorganization^2^. An abundance of research has documented the nervous system aspects of SCI and many innovative methods are being developed to treat patients affected by these devastating injuries.

It is becoming increasingly apparent that SCI also has major effects on non-nervous system tissues. SCIs disrupt the neural circuitry and signals to vital organs in the body and result in severe long-term complications outside of the central nervous system (CNS)^3^. A group of symptoms termed metabolic syndrome (MS), which include type 2 diabetes, altered body fat percentage, hypertension, and cardiovascular anomalies, have been reported in patients suffering from chronic SCI^4^. Other non-MS issues, such as lung dysfunction and autonomic dysreflexia^5,6^, osteoporosis^7^, and cardiovascular disorders, commonly affect SCI patients^8^. Cardiovascular dysfunction is the leading cause of death for those that survive the initial SCI^9,10^. Despite this, there have been relatively few studies assessing the progression of cardio-metabolic aberrations that can occur as a result of SCI.

A second factor that was included in our study was to assess sex-specific responses. SCI is more prevalent in males than females in the US, but the incidence of female injury is rising^11^. Cardio-metabolic responses are known to differ significantly between males and females in the absence of SCI^12^. Comparisons of outcome by sex are not common in SCI animal studies, but due to differences in the reproductive system, sex hormones, and chromosomal makeup between males and females, there are baseline differences in metabolism and behavior in the absence of any injury^13–15^. For example, in animal models of obesity, females are less susceptible to weight gain and hypothalamic functions differ between the sexes^16^.

To investigate these aspects, we studied the cardio-metabolic response to SCI in male and female Sprague Dawley (SD) rats. SD rats received an SCI through T10 contusion and were monitored by dual-energy X-ray absorptiometry (DEXA), echocardiography, intraperitoneal glucose tolerance tests (IPGTT), body weight, behavior assessments of functional regeneration, and histological evaluation of the heart after 20 weeks. Results revealed new aspects of cardio-metabolic alterations that occur after SCI in both males and females and, additionally, revealed aspects that were divergent between the sexes. The results support the assertion that metabolic alterations occur after SCI, can be studied using animal models, and that there is a significant difference in the development of dysfunction in females and males following SCI.

## Results

### Experimental scheme and collection of data

**Figure 1:**
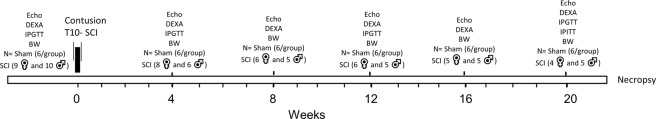
Schematic diagram of experimental design. Adult rats were used to measure a baseline for echocardiography (Echo), dual-energy X-ray absorptiometry (DEXA), intraperitoneal glucose tolerance test (IPGTT), and body weight (BW). At day 0, contusion SCI was induced, and the same measurements were taken at 4, 8, 12, 16, and 20 weeks post-injury. Intraperitoneal insulin tolerance test (IPITT) was performed only at the 20-week time point. For behavioral assessments, BBB tests were performed twice pre-injury and again at days 1, 4, 7, and then once weekly post-injury. Von Frey tests were performed once pre-injury and at days 12, 24, 35, 45, 56, 88, and 112 post-injury. At endpoint, animals were necropsied, and hearts were harvested for histological analyses.

To assess the consistency of injury caused by contusion, the spinal cords of contused animals 14 d PI were labeled for glial fibrillary acid protein (GFAP) and the size of the lesion cavity was measured with ImageJ (Supplemental Fig. 1). To ascertain cardio-metabolic and sexually dimorphic aspects of SCI, adult male and female SD rats were randomly sorted into sham or SCI groups, where there was n = 6 for female sham, n = 8 for female SCI, n = 6 for male sham, and n = 9 for male SCI at the beginning of the experiment. Animals were analyzed for parameters of body weight, DEXA, echocardiography, IPGTT, ITT, Basso, Beatie, and Bresnahan (BBB) scores, and von Frey on the schedule shown in Fig. 1. As is commonly the case, there was attrition in SCI groups with animals that died prior to the study endpoint of 20 weeks^17–19^. At the endpoint, an intraperitoneal insulin tolerance test (IPITT) was measured.

### Motor and sensory recovery following SCI

**Figure 2:**
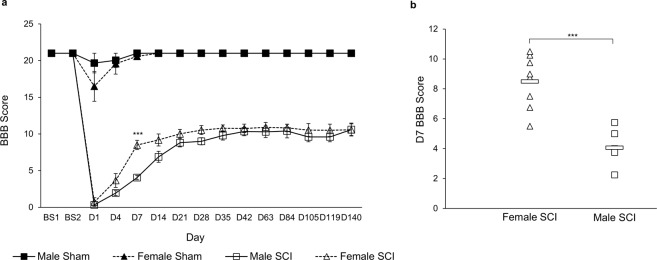
BBB scores. (**a**) BBB behavioral test performed twice before injury and on days 1, 4, 7, and then weekly post-injury. Repeated measures one-way ANOVA; data are presented as mean ± SEM (p = 0.0000255). (**b**) Scatter plot of the data from D7. Two-tailed t-test was used to examine the mean difference between Female SCI-Male SCI (n = 8 for both groups, 4.44 [2.85, 6.03], p = 0.000073).

To assess the degree of functional deficit that occurred following the SCI and the degree of recovery, BBB scores were assessed. As shown in Fig. 2, the BBB score for sham animals was not affected by the sham surgical procedure without contusion. For animals that received SCI, BBB scores dropped to near zero the first week after SCI and gradually recovered. Recovery plateaued in both male and female SCI animals at 3 weeks, consistent with previous reports^20,21^. Interestingly, some differences emerged between male and female SCI animals, where female SCI animals showed a significantly increased recovery timeline compared to males. Von Frey assessment was also performed to determine the extent of sensory function on animals (Supplemental Fig. 2). Rats of both sexes exhibited an increased threshold after SCI that returned to baseline within 24 d. Male SCI rats showed significant decrease in the threshold at 35 d in the right hind limb and a non-significant trend toward a decrease at 24, 56, and 112 d. Female sham and SCI animals were equally responsive at all tested time points. No significant differences were observed comparing the von Frey scores from male to female SCI groups.

### Echocardiography and histological analysis

**Figure 3:**
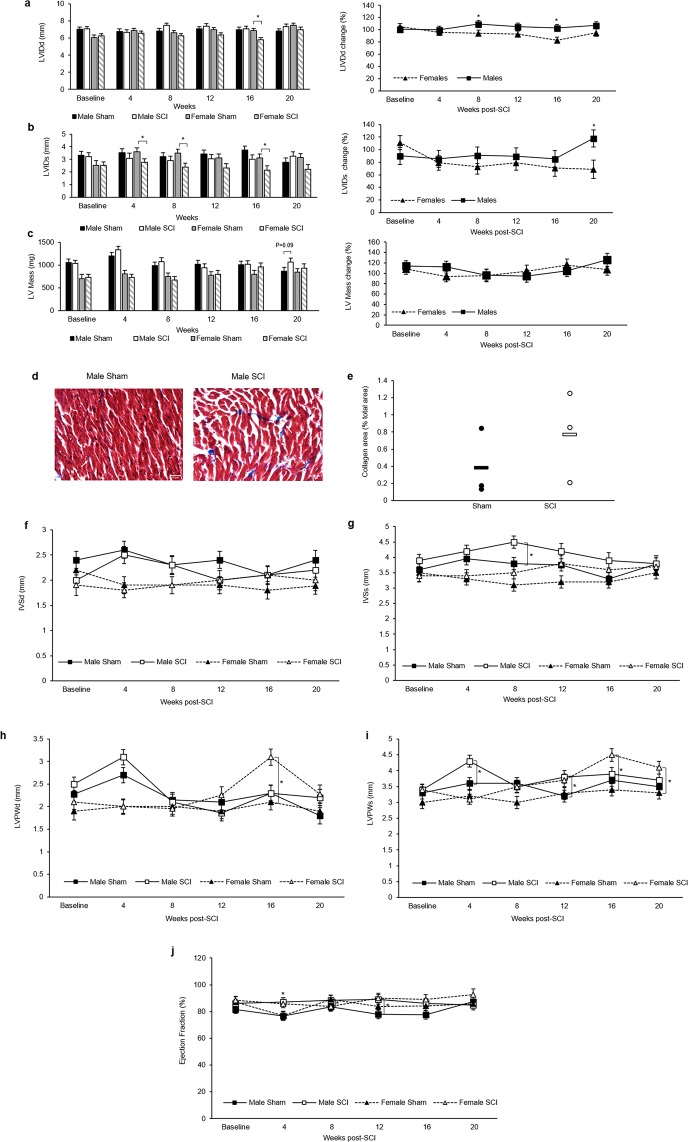
Echocardiography assessment. (**a**) Left ventricular internal diameter during diastole (LVIDd) and (**b**) during systole (LVIDs). (**c**) LV mass. (**d**) Representative 40x sections of Masson’s trichrome stained cardiac tissue from male rats. (**e**) Male collagen area (blue staining) as a % of total tissue area. (**f**) Intraventricular septum during diastole (IVSd) and (**g**) during systole (IVSs). (**h**) Left ventricular posterior wall thickness during diastole (LVPWd) and (**i**) during systole (LVPWs). (**j**) Ejection fraction (EF). Statistical analysis was performed using the repeated measures mixed procedure in SAS and data are presented as Least Squares Mean (LSM) ± SEM (*P < 0.05).

Echocardiography was performed to assess cardiac structure and function. LVID during systole (LVIDs) was reduced significantly in SCI females at 4-, 8-, and 16-weeks PI and trended to decrease at 12- and 20-weeks PI. No significant reduction in LVIDs between male sham and SCI was observed. When comparing the percent LVID change between male and female SCI animals, the data indicates that the percent diameter change for female SCI animals was greater than what was observed for male SCI animals, as compared to sham (Fig. 3a,b). Measurements of LV mass indicated no change for females but a trend of increased LV mass in SCI males at 20 weeks compared to sham (Fig. 3c). Masson’s trichrome staining of left ventricular sections showed no significant change in collagen accumulation in SCI females and a trend towards increase in SCI males as compared to their sham controls (Fig. 3d,e). No significant differences were observed in IVS thickness during diastole (IVSd) in both SCI males and SCI females compared to their sham control. However, IVS thickness during systole (IVSs) significantly increased in SCI males at 8 weeks PI compared to sham males (Fig. 3f,g). LVPW thickness during diastole (LVPWd) increased significantly in SCI females at 16 weeks PI but not in SCI males. LVPW thickness during systole (LVPWs) increased significantly in SCI males at 4- and 12-weeks PI and in SCI females at 16- and 20-weeks PI (Fig. 3h,i). Ejection fraction (EF) percentage also significantly increased in SCI males at 4- and 12-week PI and in SCI females at 4- and 8-weeks PI (Fig. 3j).

### Animal weight and body composition

**Figure 4:**
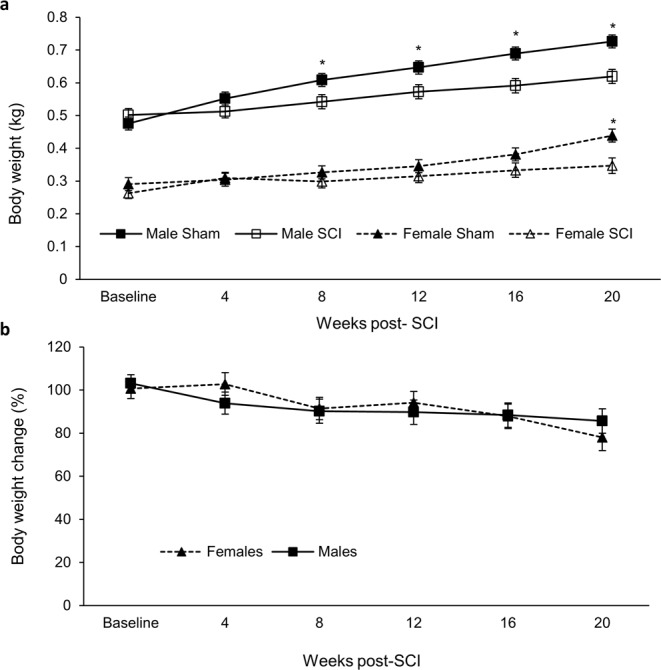
(**A**) Animal body weight. (**B**) Percent weight change between male and female SCI. Statistical analysis was performed using the repeated measures mixed procedure in SAS and data are presented as Least Squares Mean (LSM) ± SEM (*P < 0.05).

At 8-weeks PI, the body weight of SCI males was significantly reduced compared to male sham animals and this continued until the study endpoint (Fig. 4a). Female SCI animals similarly did not gain weight at the same rate as their sham counterparts over the course of the 20-week study, but differences only became significant at 20 weeks (Fig. 4a). When comparing the percent weight change between male and female SCI animals, the data indicates no significant differences observed among the groups (Fig. 4b).

**Figure 5:**
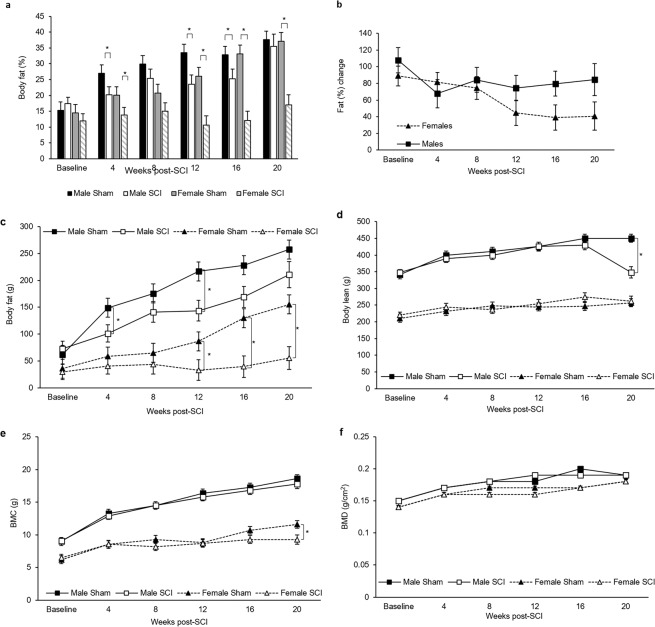
(**a**) Animal body fat. (**b**) Percent weight change between male and female SCI. (**c**) Body fat, (**d**) body lean, (**e**) bone mineral density (BMC), and (**f**) bone mineral content (BMD). Statistical analysis was performed using the repeated measures mixed procedure in SAS and data are presented as Least Squares Mean (LSM) ± SEM (*P < 0.05).

For DEXA measurements of body composition, several distinctions were noted. Prior to SCI, the total body fat (Fig. 5c) and percentage of body fat did not differ between male animals or female animals (Fig. 5a). By 4-weeks PI, however, total body fat percentage differed significantly between shams and SCIs by sex (Fig. 5a). This was most pronounced for female SCI over the course of the study as all other groups, including male SCI, showed increasing body fat percent over time. This corresponded to a significant change in percent body fat for female SCI compared to female sham (Fig. 5b). Other parameters of body composition such as lean muscle mass, bone mineral content, and bone mineral density did not differ between groups (Fig. 5d–f).

### Glucose and insulin metabolism

**Figure 6:**
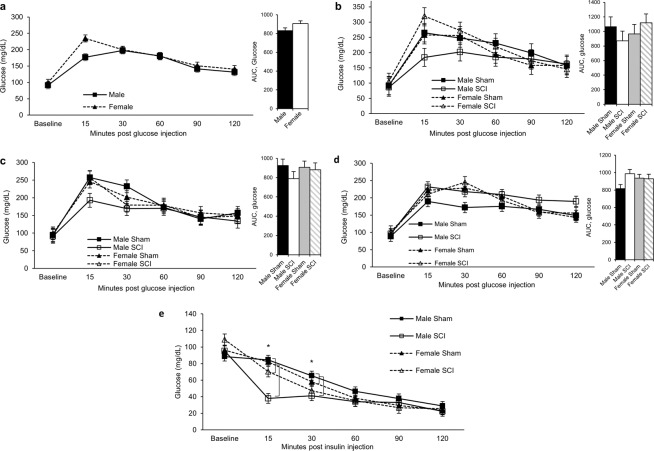
IPGTT at different time points. (**a**) IPGTT pre-injury, (**b**) at 4 weeks, (**c**) at 12 weeks, and (**d**) at 20 weeks PI. (**e**) IPITT at different time points. Statistical analysis was performed using the repeated measures mixed procedure in SAS and data are presented as Least Squares Mean (LSM) ± SEM (*P < 0.05).

IPGTTs showed glucose concentrations and area under the curve (AUC) were similar between males and females pre-injury (Fig. 6a). No significant differences were observed in glucose concentration and AUC at 4-, 12-, and 20-weeks PI for SCI animals compared to sham (Fig. 6b–d). Fasting glucose concentrations were similar between the animals up to 20-weeks PI (Supplemental Fig. 3). To examine glucose concentrations in response to insulin, IPITT was performed at 20-weeks PI, several days after the final IPGTT. Interestingly, male SCI animals experienced a more rapid decline in blood glucose following insulin administration than other groups, suggesting a heightened response to insulin (Fig. 6e). At 15 minutes post glucose administration, blood glucose in male SCI animals was significantly lower than all other groups. At 30 minutes post administration, male SCI glucose concentrations differed significantly from male and female sham groups.

## Discussion

The trauma induced by SCI affects spinal pathways that are essential not only for somatic but also for the function of the autonomic nervous system. Where the majority of SCI research has understandably focused on aspects associated with motor and sensory conduction, it is becoming increasingly apparent that SCI also induces a host of changes that are due either to primary damage to pathways that influence autonomic activity or are secondary to sensorimotor impairment^22^. For many of the processes that fall under autonomic function, such as metabolism and cardiovascular function, sexual dimorphism has been documented but not in the context of SCI^12–14^. To investigate the potential for cardio-metabolic and sexual dimorphism in SCI, we studied the cardio-metabolic effects on adult male and female rats for 20 weeks, spanning the acute and chronic phases after SCI^23^.

The primary advancements of these data are to expand upon the known sensorimotor and cardio-metabolic effects of SCI and show how many effects of SCI can differ based on sex. Regarding sensorimotor function, BBB test indicated a more rapid recovery timeline for females than males with SCI (Fig. 2). This is consistent with previous reports that have shown improved BBB recovery in female animals. Hauben *et al*. performed mild and severe contusions in rats at T8 and found that by 3 weeks females had recovered significantly better than males, and maintained this throughout the 120 d study^24^. Datto *et al*. also performed a moderate contusion at T8 and results showed that by 5 weeks females had recovered significantly more than males and maintained this for duration of the 13-week study^25^. Both studies used more animals, with Datto *et al*. using substantially more, and performed their SCI higher up the spinal cord (at T8 as opposed to T10). While female SCI rats reached a higher BBB score more quickly in our study, the difference was not sustained as it was in these two studies. Von Frey assessment of sensory function was largely comparable for both sexes, but males did exhibit an indication of mechanical allodynia (Supplemental Fig. 2). These differences occurred with the same method of injury despite significant initial differences in weight between males and females (Fig. 4a).

In general, human clinical studies assessing functional outcomes that have included sex as a variable have been mixed. Studies by Greenwald *et al*. and Scivoletto *et al*. reported no significant alterations in functional outcomes at discharge^26,27^, whereas Sipski *et al*. observed improved sensorimotor outcomes for females one-year post SCI compared to males as measured by the American Spinal Injury Association (ASIA) metrics^28^. A key variable unaccounted for in these studies is the extent of physical rehabilitation the patients received. The rats in our study received no rehabilitative therapy, suggesting that baseline female recovery may be more rapid than in males and that females may be less prone to the development of mechanical allodynia.

For cardiac function, it is well established that male rats with SCI exhibit alterations in cardiac structure and function largely consistent with the results of our study, albeit using different injury models and a shorter timeline of analysis^29–33^. In comparing the sexes, the results here show males exhibited a slight increase in LVID while females exhibited a reduction (Fig. 3a,b). This LVIDs reduction in SCI females was associated with an increase in LVPW thickness only at 16- and 20-weeks PI (Fig. 3h,i). Such ventricular remodeling can be adaptive or maladaptive^34^. Masson’s trichrome staining suggested cardiac fibrosis was a risk for male but not female SCI animals but did not rise to statistical difference (Fig. 3d,e**)**. A study by Cragg *et al*., however, showed a significant increase in cardiovascular dysfunction after SCI, but the contributions of sex to dysfunction in this study was considered a confounding variable due to the known differences in the risk for developing cardiovascular dysfunction between uninjured males and females^35,36^. Our data show that female animals do developed cardiovascular alterations as a result of SCI and suggests that they are adaptive whereas male alterations may be more prone to being maladaptive.

From the DEXA measurements, SCI caused significant differences in body mass (Fig. 4). In females, this appears to be primarily caused by alterations in body fat as female SCI rats did not gain any body fat at 20 weeks PI compared to baseline (Fig. 5a). This contrasts for female sham, male sham, and male SCI that respectively showed 156, 146, and 105% increases in body fat at 20 weeks compared to pre-injury. Female SCI BMC was significantly decreased at 20 weeks compared to sham but did not correlate with a decrease in BMD, indicating a likely decrease in overall bone content but not density in female SCI rats (Fig. 5e,f)^37^. Total lean muscle mass did not differ between groups.

The lack of any fat gain in female SCI is intriguing. Common reasons for this outside of an SCI would include increased exercise, altered metabolism, and lower foot intake. Our study did not monitor activity nor dietary intake, which would be a useful addition for future studies. It is also not known whether SCI females failed to generate new fat or if they more rapidly metabolized fat as an energy source. Fasting glucose and AUC for the IPGTT indicated no difference from female sham to SCI (Supplement Figs 3 and 6), but many other factors could be contributing to this distinct divergence in fat accumulation.

This data from DEXA measurements somewhat diverge from what is available from human clinical studies, that show human SCI patients gain fat and lose increased lean muscle mass, BMD, and BMC PI^38–40^. While female SCI animals began to show a difference in BMC, this divergence may indicate a limitation of the rat model to recapitulate these defects but also may be due to the difference in the degree of injury. The aforementioned studies included many different injury types of differing severity whereas all rats used in this study would be classified incomplete paraplegia, showing full restoration of von Frey responses and partial hind limb motor recovery by the BBB within weeks of injury (Fig. 2, Supplemental Fig. 2). Exercise post SCI is known to prevent muscle atrophy and reduces bone loss in human SCI patients^41,42^.

While the animal numbers in this pilot study were sufficient for statistical comparisons reported between groups, attrition of animals over the course of the study is a potential limitation. Attrition in animal survival after SCI occurs despite careful intensive care and monitoring. Comparable rates of attrition have been noted for contusions similar in severity to that of our study (Fig. 1)^17,18^. Future studies assessing longer-term time points in animals with severe SCIs would benefit from inclusion of more animals and the development of improved protocols to mitigate attrition.

## Materials and Methods

### Animal use and procedure for SCI

All animal use and procedures were performed with approval by the University of Wyoming Institutional Animal Use and Care Committee. Animals were cared for in accordance with the Guide for the Care and Use of Laboratory Animals^43^. Adult male and female SD rats were purchased from Charles River (Wilmington, Massachusetts) and randomly assigned to one of two treatment groups: sham or SCI. SCI was induced by contusion to the spinal cord as previously described^20^. Briefly, rats were anesthetized by 2% isoflurane inhalation and the surgical site at T10 was shaved and sterilized by iodine and isopropyl alcohol. The animal was draped, the surgical site was incised, and a partial dorsal laminectomy was performed at T10 to expose the spinal cord without penetrating the dura. To generate the SCI, a 10 g rod was dropped from a height of 35 mm onto the exposed spinal cord using an NYU-type impactor, where the rod remained in contact with the spinal cord for 3 s before removal. A small piece of adipose tissue was placed on top of the laminectomy site and the musculature and skin were sutured and closed with 6-0 sutures and wound clips. Sham animals received the same procedure and surgery of SCI groups but did not receive an impact. After surgery, animals received buprenorphine (0.05 mg/kg, Par Pharmaceutical Chestnut Ridge, NY 10977) twice daily for 3 d, and baytril (5 mg/kg/day, Bayer Healthcare LLC, Animal Health Division Shawnee Mission, Kansas 66201 USA) once daily for 7 d to prevent secondary infection. Bladders were expressed twice daily until animals were observed to have recovered bladder function.

### Histological assessments of injury

To assess the degree of injury caused by impact, spinal cords were harvested from contused male animals at 14 d PI. Animals were anesthetized with inhalation of 2% isoflurane with pure oxygen and were euthanized via perfusion with 4% paraformaldehyde (PFA) in 1x phosphate buffer solution (PBS) (Cat. No. 14200075, Life Technologies). Samples were placed in OCT compound (Cat. No. 625501-01, Sakura Finetek USA, Inc.) and longitudinal sections of 20 µm were sliced using a cryostat (Leica Biosystems CM3050 S). Fixed sections of spinal cord tissue were washed with 1x PBS 3x for 5 minutes each, then blocked using AB media (10% BSA, 1% normal goat serum, 0.3% Triton X-100 in 1x PBS) for 1 h. Sections were incubated with primary anti-glial fibrillary acidic protein (GFAP) antibody (1:500, chicken IgY, Cat. No. AB5541, Millipore), diluted in AB media (1% BSA, 0.1% normal goat serum, 0.3% Triton X-100 in 1x PBS) at 4 °C overnight, washed 3x for 5 minutes each, and incubated with secondary antibody, Alexa flour 555 goat anti-chicken IgY antibody (1:400, Cat. No. A21437, ThermoFisher Scientific), in AB media (1% BSA, 0.1% normal goat serum, 0.3% Triton X-100 in 1x PBS) for 2 h. After being washed 3x for 5 minutes each, slides were covered with mounting media and cover slipped (Cat. No. F6182, Sigma). Sections were imaged on a confocal microscope (Zeiss) and ImageJ software (NIH) was used to measure size of lesion cavity. A minimum of 3 sections were averaged to represent each animal, with 6 animals in total to assess consistency of SCI.

### Assessment of functional regeneration

Injury and functional recovery post-injury (PI) were assessed using the Basso, Beattie, and Bresnahan scale (BBB) and von Frey tests. BBB was performed as previously described^20,21^: each rat was placed in a small plastic pool and was scored by two independent observers during a 4-minute session, assigning scores from 0–21 to each hind limb, where 21 stands for perfectly normal, and 0 as completely paralyzed. The average score of the two observers was the final score of that individual animal and the average score of all animals within a group is considered the final score for that group.

Von Frey tests were performed as previously described^44,45^. Briefly, rats were placed in small individual cages with mesh wire at the bottom. Von Frey filaments (4, 6, 8, 10, 15, 26, 60, 100, 180, and 300 g) were applied perpendicularly on the plantar surface of the front left, hind left, and hind right paws of each rat. The lightest filament was used first, incrementing to the heaviest filament. Each filament was performed on each paw 10 times. The filament was considered as threshold if a rat responded more than 5 times out of 10.

### Echocardiography

Echocardiography imaging and analysis were used to asses cardiac structure and function following SCI at different time points as we previously described^46^. Rats were anesthetized with isoflurane (induction at 3% and maintained on 1.5%) and heart rate was maintained between 300 and 350 beats per minutes. The hair was removed from the anterior chest wall and ultrasound transmission gel was applied. To visualize the cross-sectional view of the left ventricle, a linear transducer was used to examine the parasternal short axis view. Cine-loop analysis was performed to examine the systolic and diastolic phases of the heart. M-mode echocardiograph was acquired using echocardiography imaging equipment (VisualSonics Vevo 2100, FUJIFILM). The measurement was performed by tracing the internal diameters of the ventricle, averaged from three consecutive cycles for each animal. Several cardiac parameters were assessed, including left ventricle internal diameter (LVID), left ventricular (LV) mass, intraventricular septum (IVS), left ventricular posterior wall thickness (LVPW), and ejection fraction (EF).

### Animal weight and body composition

DEXA scans were used to asses body fat percentage, lean tissue, and bone mineral density and content as previously described^47^. Briefly, animals were anesthetized by isoflurane inhalation and positioned prone on the DEXA scanner (GE Lunar Prodigy 8743; Madison, WI). A low-level pencil-beam X-ray moved transversely from the head to the tail across the sedated animal. Differences in absorbance of X-ray was determined according to tissue density and fat percentage was calculated using fat and body mass^48^. Animals were weighed at each time point.

### Glucose and insulin metabolism

IPGTT was performed to assess glucose metabolism using published protocols^49^. Rats were fasted for 16 h and weighed. Blood was sampled from the lateral tail vein using 24-g needles. Glucose levels in blood were quantified using a glucose meter (Bayer Contour Next EZ blood glucose meter, Bayer HealthCare, IN) before and after intraperitoneal administration of 20% (2 g/kg) dextrose (Sigma. D9434) at 15, 30, 60, 90, and 120 min. IPITT was performed where insulin (0.75 IU/kg, Humulin R, Eli Lilly, Indianapolis, IN) was administered by intraperitoneal injection and glucose concentrations were quantified similarly to IPGTT.

### Histological analysis of myocardial fibrosis

Hearts were removed and fixed in 10% formalin for 48 hours, dehydrated with ethanol, and embedded in paraffin. Sections were cut at 7 µm, with 50 µm between each section, and Masson’s trichrome staining was performed (Sigma-Aldrich, HT15-1KT) according to the manufacturer’s instructions. Analysis and quantification of fibrosis was performed as previously described^46,50^, where 3 sections per heart were imaged on an Olympus BX51 microscope and quantified using ImageJ (NIH, Bethesda, MD).

### Statistical analysis

Statistical analysis was performed using the mixed procedure in SAS (SAS Institute, Cary NC, USA) using fixed effects group*time*sex (repeated measures), repeated measures one-way analysis of variance (ANOVA), or unpaired two-tailed t-test. A p-value of <0.05 was considered significant. Data are presented as the mean, or least squares means (LSM), ± standard error of the mean (SEM).

## Supplementary information

Supplementary figure 1.

Supplementary figure 2.

Supplementary figure 3.

Supplementary figure legend.

## Data Availability

The datasets generated and/or analyzed during the current study are available from the corresponding author upon reasonable request.
