# Supplementary figures and images for: A Long-Term Pilot Study on Sex and Spinal Cord Injury Shows Sexual Dimorphism in Functional Recovery and Cardio-Metabolic Responses
Source: Sci Rep. 2020 Feb 17;10:2762. doi: 10.1038/s41598-020-59628-6 (PMC7026076; doi:10.1038/s41598-020-59628-6)

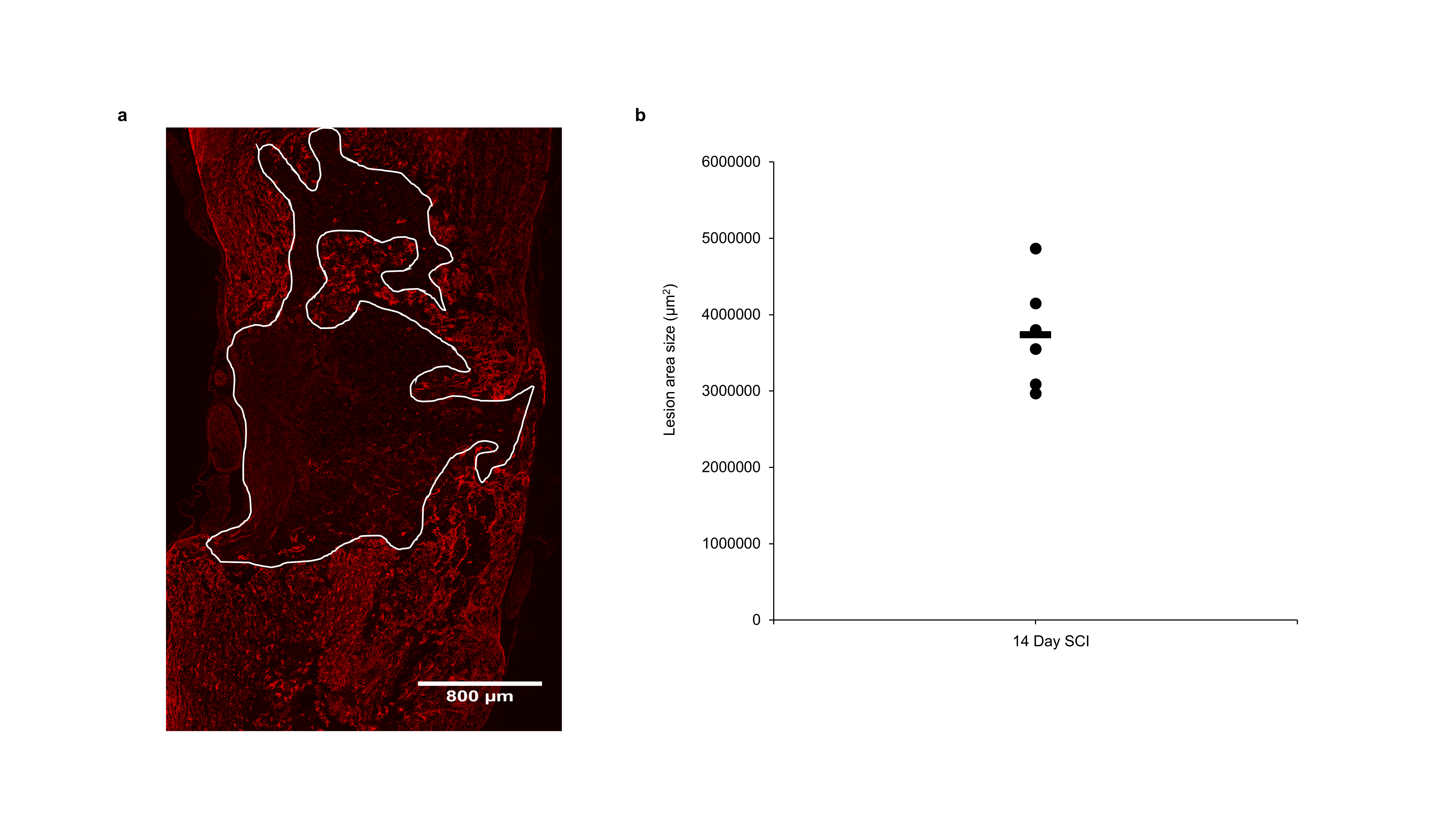

Supplement: Supplementary file 1 — Supplementary figure 1. [file 41598_2020_59628_MOESM1_ESM.tif]

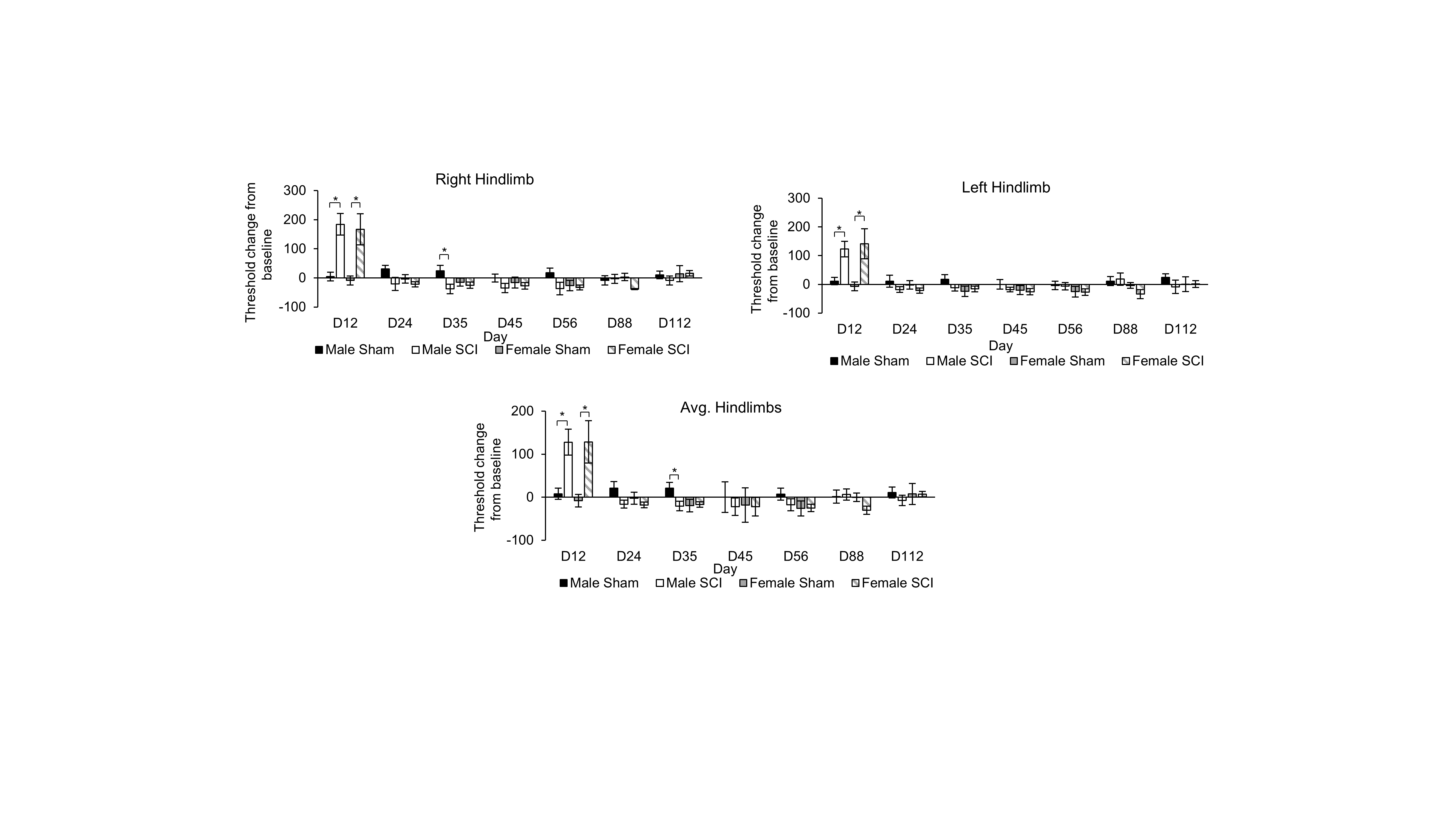

Supplement: Supplementary file 2 — Supplementary figure 2. [file 41598_2020_59628_MOESM2_ESM.tif]

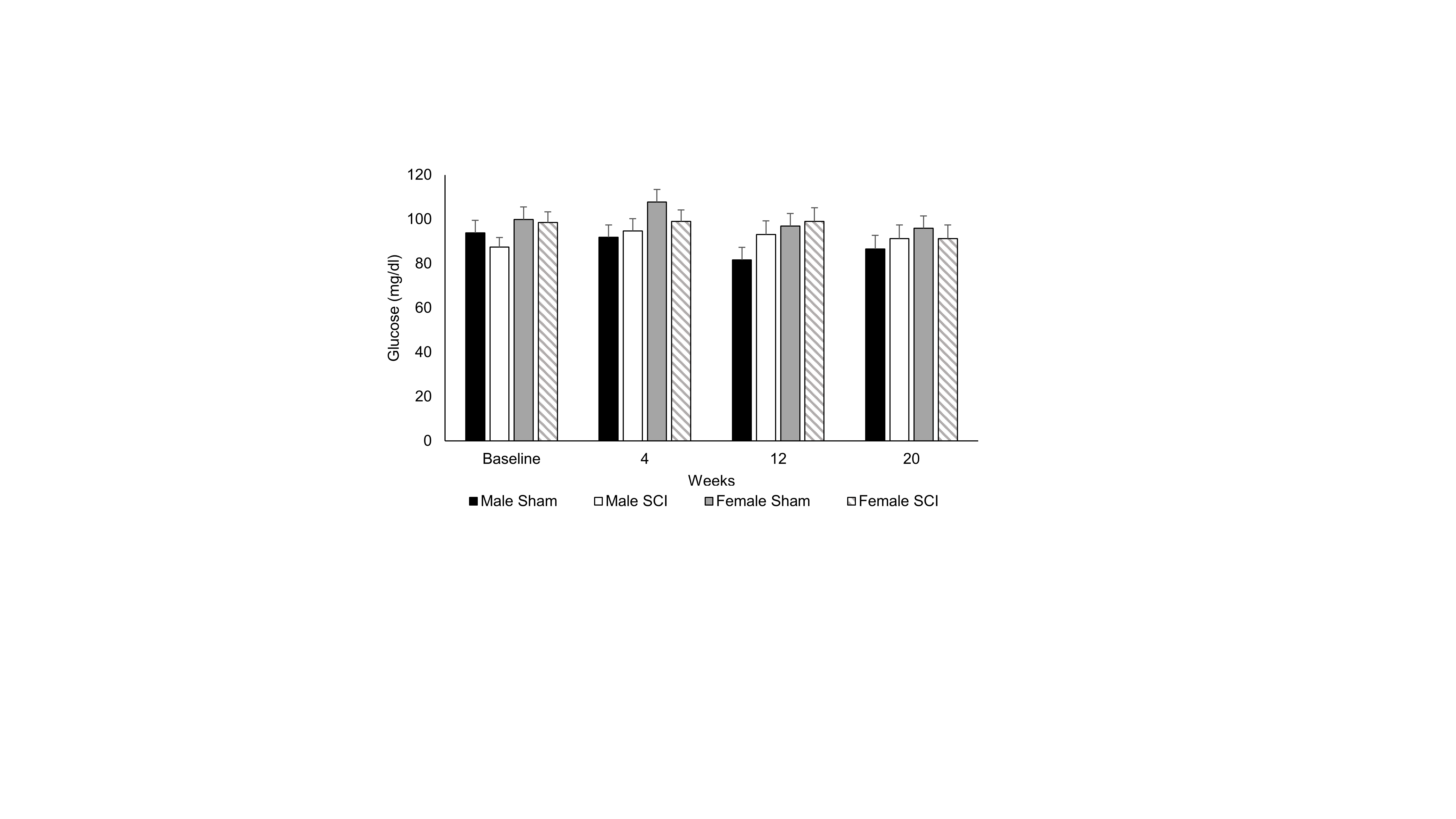

Supplement: Supplementary file 3 — Supplementary figure 3. [file 41598_2020_59628_MOESM3_ESM.tif]
