# Supplementary material for: A Long-Term Pilot Study on Sex and Spinal Cord Injury Shows Sexual Dimorphism in Functional Recovery and Cardio-Metabolic Responses
Source: Sci Rep. 2020 Feb 17;10:2762. doi: 10.1038/s41598-020-59628-6 (PMC7026076; doi:10.1038/s41598-020-59628-6)
Supplement: Supplementary file 4 — Supplementary figure legend. [file 41598_2020_59628_MOESM4_ESM.docx]

A Long-Term Pilot Study on Sex and Spinal Cord Injury Shows Sexual Dimorphism in Functional Recovery and Cardio-Metabolic Responses

Adel B. Ghnenis^1^, Daniel T. Burns^1^, Wupu Osimanjiang ^1^, Guanglong He^1^, Jared S. Bushman^1^*

^1^University of Wyoming School of Pharmacy, 1000 East University Avenue, Dept. 3375, Laramie, WY, USA, 82071

Adel B. Ghnenis, Ph.D., Post-doctoral Fellow. Texas A&M Health Science Center. 4100 Medical Research and Education Building 8447 Riverside Parkway | Bryan, TX 77807 – USA, 307-399-868, ghnenis@tamu.edu

Daniel Burns, M.S., Ph.D. Candidate. 1000 E. University Ave, Dept 3375, Laramie WY, USA, 82071. 307-766-6185, dburns5@uwyo.edu

Wupu Osimanjiang, M.S., Ph.D. Candidate. 1000 E. University Ave, Dept 3375, Laramie WY, USA, 82071. 307-766-6185, owupu@uwyo.edu

Guanglong He, Ph.D., Associate Professor. 1000 E. University Ave, Dept 3375, Laramie WY, USA, 82071. 307-766-6637, ghe@uwyo.edu

Jared S. Bushman, Ph.D., Assistant Professor. 1000 E. University Ave, Dept 3375, Laramie WY, USA, 82071. 307-766-4198, jbushman@uwyo.edu *To whom correspondence should be addressed.

**Supplemental Figure 1** Spinal cord injury assessment. **A** Representative glial fibrillary acidic protein (GFAP) stained section 14 d PI showing lesion cavity (outlined in white). **B** Scatter plot of lesion size at injury site (n = 6).

**Supplemental Figure 2** Von Frey scores. Von Frey is performed once before the injury, and at different time points post-injury. For each time point, mechanical threshold is measured and difference with baseline is calculated. Repeated measures one-way ANOVA; data are presented as mean ± SEM (* P < 0.05).

**Supplemental Figure 3** Fasting glucose concentrations at different time points. Statistical analysis was performed using the repeated measures mixed procedure in SAS and data are presented as Least Squares Mean (LSM) ± SEM.
